# Uma Apresentação Atípica e Não-Cardíaca de Cardiomiopatia Hipertrófica

**DOI:** 10.36660/abc.20220933

**Published:** 2023-06-16

**Authors:** André Alexandre, Carla Roque, Isabel Sá, João Silveira, Severo Torres

**Affiliations:** 1 Centro Hospitalar Universitário do Porto EPE Porto Portugal Centro Hospitalar Universitário do Porto EPE, Porto – Portugal; 2 Universidade do Porto Instituto de Ciências Biomédicas Abel Salazar Porto Portugal Universidade do Porto – Instituto de Ciências Biomédicas Abel Salazar (ICBAS), Porto – Portugal

**Keywords:** Cardiomiopatia Hipertrófica, Acidente Vascular Cerebral, Aneurisma Cardíaco, Imagem Multimodal

Um homem brasileiro de 33 anos foi admitido no departamento de emergência com início súbito de afasia global e hemiparesia direita. Uma angiotomografia de urgência das artérias cerebrais confirmou o diagnóstico de acidente vascular cerebral (AVC), mostrando uma oclusão da artéria carótida interna esquerda. O paciente foi submetido a trombólise sistêmica e trombectomia mecânica com posterior melhora neurológica. Considerando o diagnóstico de AVC em um adulto jovem provavelmente de origem cardioembólica, ele realizou uma avaliação diagnóstica completa. De realçar que tinha antecedentes familiares de cardiomiopatia, nomeadamente o pai com cardiomiopatia hipertrófica (CMH) e o avô com doença de Chagas. No entanto, o paciente não apresentava sintomas cardíacos (como dispneia aos esforços, dor torácica, palpitações ou síncope), fatores de risco cardiovascular ou história de abuso de drogas ilícitas, e não fazia exames cardíacos desde a juventude. Durante a internação, seu eletrocardiograma apresentava ritmo sinusal (68/minutos) com inversão da onda T nas derivações inferiores (II, III, aVF), derivações I e V6, mas sem critérios de hipertrofia ventricular esquerda (VE). Realizou também Holter 24 horas, descartando fibrilação atrial ou outras arritmias. A ecocardiografia transtorácica ( Figura 1 , Vídeo Complementar 1, Vídeo Complementar 2) revelou hipertrofia septal ventricular assimétrica moderada (espessura do septo interventricular 14 mm, espessura da parede posterior 9 mm), fração de ejeção VE discretamente reduzida (45%), acinesia apical e imagem sugestivo de trombo, explicando o AVC cardioembólico. A ressonância magnética cardíaca (RMC) confirmou o diagnóstico de CMH, com extensa fibrose apical e acinesia dos segmentos apicais, delineando aneurisma apical e trombo ( Figura 2 ). A coronariografia foi realizada quanto à possibilidade de cardiopatia isquêmica concomitante, descartando-se doença arterial coronariana obstrutiva. O exame sorológico para *Trypanosoma cruzi* foi negativo. O paciente iniciou anticoagulação sistêmica com antagonista da vitamina K (AVK). Considerando o diagnóstico de CMH com fibrose apical extensa e aneurisma apical, após tomada de decisão compartilhada pelo paciente, optou-se pela colocação de cardioversor-desfibrilador implantável subcutâneo (S-CDI). Teve alta após 18 dias. No seguimento de 1 ano, o ecocardiograma transtorácico de controle mostrou resolução completa do trombo apical, e o paciente permaneceu em anticoagulação sistêmica com AVK.

**Figura 1:**
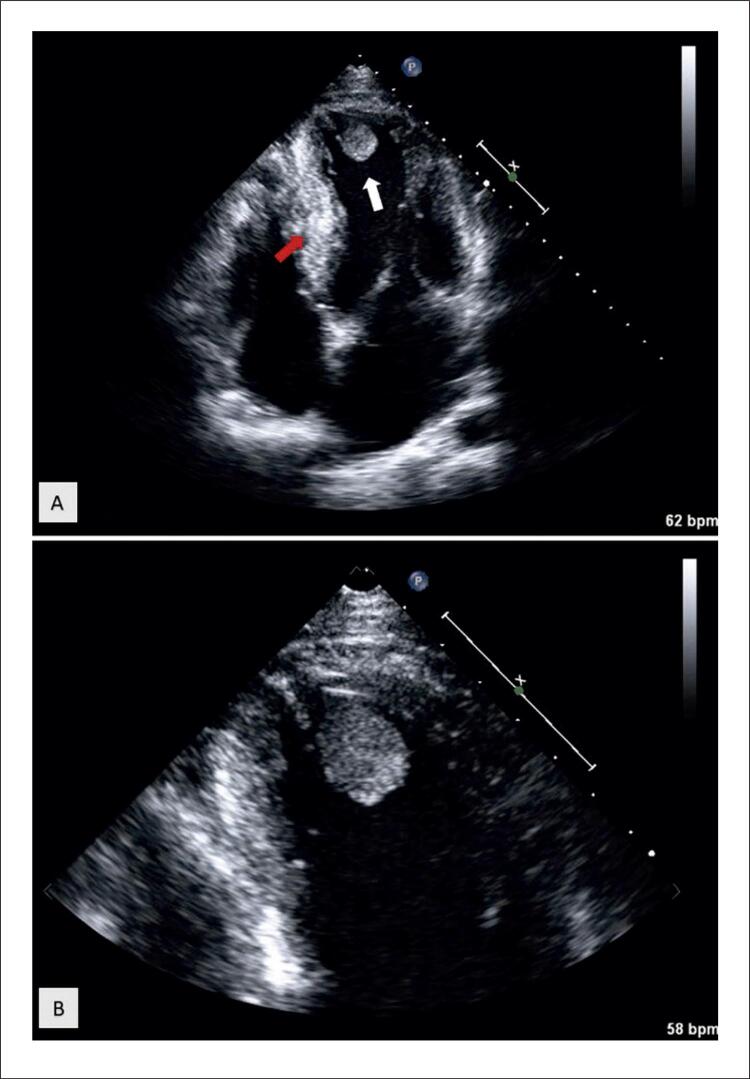
– Ecocardiografia transtorácica levantando a suspeita de cardiomiopatia hipertrófica e trombo apical. Painel A) Ecocardiograma transtorácico (corte apical quatro câmaras) mostrando hipertrofia septal assimétrica do ventrículo esquerdo (VE) (seta vermelha) e imagem sugestiva de trombo (seta branca). Painel B) imagem de zoom (da visão apical de quatro câmaras) representando uma massa hiperecóica dentro do ápice do VE. VE: ventrículo esquerdo.

**Figura 2:**
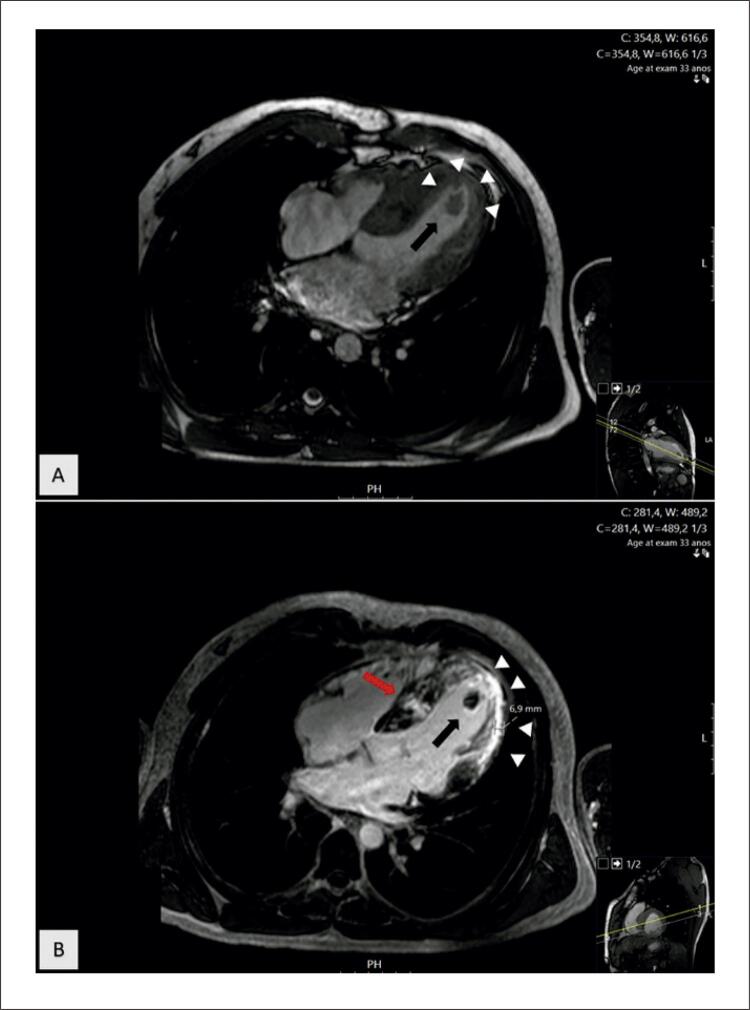
– Ressonância magnética cardiovascular (RMC) confirmando o diagnóstico de cardiomiopatia hipertrófica. Painel A) Imagem de RMC sem contraste em precessão livre de estado estacionário (visão de quatro câmaras) mostrando hipertrofia significativa do ventrículo esquerdo (VE) e um ápice aneurismático do VE (setas) contendo um trombo intracavitário (seta preta). Painel B) Sequência de recuperação da inversão da RMC (visão de quatro câmaras) confirmando o diagnóstico de cardiomiopatia hipertrófica. Há realce tardio intramiocárdico irregular (RTG) no septo interventricular (seta vermelha), RTG transmural no ápice e segmento apical lateral do VE (setas brancas) e um trombo intracavitário (seta preta) dentro do aneurisma do VE.

A CMH é uma doença cardíaca relativamente comum, mas ainda subdiagnosticada.^1^ Este caso ilustra uma primeira apresentação não cardíaca extremamente rara e com risco de vida de CMH. Esse fenótipo incomum de CMH com aneurisma apical do VE com cicatriz e paredes finas está associado a um risco aumentado de morte súbita arrítmica e acidente vascular cerebral tromboembólico.^2 , 3^ A imagiologia cardiovascular multimodal é de suma importância para o diagnóstico etiológico de acidente vascular cerebral cardioembólico em uma idade jovem. A penetração ampliada da RMC na prática de rotina é essencial para diagnosticar esse fenótipo de CMH, que traz implicações prognósticas e de manejo significativas, como terapia com cardioversor desfibrilador implantável e anticoagulação sistêmica para prevenção de AVC.^2 , 4^

## * Material suplementar

Para informação adicional, por favor, clique aqui.

Para assistir ao vídeo suplementar 1, por favor, clique aqui.

Para assistir ao vídeo suplementar 2, por favor, clique aqui.
