# Supplementary material for: Uma Apresentação Atípica e Não-Cardíaca de Cardiomiopatia Hipertrófica
Source: Arq Bras Cardiol. 2023 Jun 16;120(6):e20220933. [Article in Portuguese] doi: 10.36660/abc.20220933 (PMC10344080; doi:10.36660/abc.20220933)
Supplement: Translation

# Translation: An Atypical Non-Cardiac Presentation of Hypertrophic Cardiomyopathy

**Authors:** André Alexandre, Carla Roque, Isabel Sá, João Silveira, Severo Torres

**Affiliations:** 1 Portugal Centro Hospitalar Universitário do Porto EPE, Porto – Portugal; 2 Portugal Universidade do Porto – Instituto de Ciências Biomédicas Abel Salazar (ICBAS), Porto – Portugal

**Keywords:** Hypertrophic Cardiomyopathy, Stroke, Heart Aneurysm, Multimodal Imaging

A 33-year-old Brazilian man was admitted to the emergency department with sudden onset of global aphasia and right hemiparesis. An urgent CT angiography of the cerebral arteries confirmed the stroke diagnosis, showing an occlusion of the left internal carotid artery. The patient underwent systemic thrombolysis and mechanical thrombectomy with subsequent neurological improvement. Considering the stroke diagnosis in a young adult most likely of cardioembolic origin, he undertook a thorough diagnostic workup. Of importance, he had a family history of cardiomyopathy, namely his father with hypertrophic cardiomyopathy (HCM) and his grandfather with Chagas disease. However, the patient had no cardiac symptoms (such as exertional dyspnea, chest pain, palpitations, or syncope), cardiovascular risk factors, or history of illicit drug abuse, and he had not performed heart tests since his youth. During hospitalization, his electrocardiogram showed sinus rhythm (68/minute) with T-wave inversion in inferior leads (II, III, aVF), leads I and V6, but no criteria for left ventricular (LV) hypertrophy. He also performed a 24-hour Holter monitoring, ruling out atrial fibrillation or other arrhythmias. Transthoracic echocardiography ( Figure 1 , Supplementary Video 1, Supplementary Video 2) revealed moderate asymmetric septal LV hypertrophy (interventricular septum thickness 14 mm, posterior wall thickness 9 mm), mildly reduced LV ejection fraction (45%), apical akinesia, and an image suggestive of thrombus, explaining the cardioembolic stroke. Cardiovascular magnetic resonance (CMR) confirmed the diagnosis of HCM, with extensive apical fibrosis and akinesia of the apical segments, outlining an apical aneurysm and thrombus ( Figure 2 ). Coronary angiography was performed regarding the possibility of concomitant ischemic heart disease, ruling out obstructive coronary artery disease. Serologic examination for *Trypanosoma cruzi* was negative. The patient was started on systemic anticoagulation with a vitamin K antagonist (VKA). Considering the diagnosis of HCM with extensive apical fibrosis and an apical aneurism, after patient-shared decision-making, it was decided to place a subcutaneous implantable cardioverter-defibrillator (S-ICD). He was discharged after 18 days. At the 1-year follow-up, control transthoracic echocardiography showed complete resolution of the apical thrombus, and the patient remained on systemic anticoagulation with a VKA.

**Figure 1:** – Transthoracic echocardiography raising the suspicion of hypertrophic cardiomyopathy and apical thrombus. Panel A) Transthoracic echocardiography (apical four-chamber view) showing asymmetric septal left ventricular (LV) hypertrophy (red arrow) and an image suggestive of thrombus (white arrow). Panel B) Zoom image (from apical four-chamber view) depicting a hyperechoic mass within the LV apex.

**Figure 2:** – Cardiovascular magnetic resonance (CMR) confirming the diagnosis of hypertrophic cardiomyopathy. Panel A) Cine steady state-free precession non-contrast CMR image (four-chamber view) showing significant left ventricular (LV) hypertrophy and an aneurysmatic LV apex (arrowheads) containing an intracavitary thrombus (black arrow). Panel B) CMR inversion recovery sequence (four-chamber view) confirming the diagnosis of hypertrophic cardiomyopathy. There is patchy intramyocardial late gadolinium enhancement (LGE) in the interventricular septum (red arrow), transmural LGE at the apex and lateral apical LV segment (white arrowheads), and an intracavitary thrombus (black arrow) within the LV aneurysm.

HCM is a relatively common but still underdiagnosed cardiac disease.^1^ This case illustrates an extremely rare and life-threatening non-cardiac first presentation of HCM. This unusual HCM phenotype with a thin-walled, scarred LV apical aneurysm is associated with an increased risk of sudden arrhythmic death and thromboembolic stroke.^2 , 3^ Multimodality cardiovascular imaging is of paramount importance for the etiological diagnosis of cardioembolic stroke at a young age. The expanded penetration of CMR into routine practice is essential to diagnose this HCM phenotype, which raises significant prognostic and management implications, such as implantable cardioverter defibrillator therapy and systemic anticoagulation for stroke prevention.^2 , 4^

## * Supplemental Materials

For additional information, please click here.

See the Supplemental Video 1, please click here.

See the Supplemental Video 2, please click here.
